# Systemic chemotherapy with doxorubicin, cisplatin and capecitabine for metastatic hepatocellular carcinoma

**DOI:** 10.1186/1471-2407-6-3

**Published:** 2006-01-05

**Authors:** Se Hoon Park, Yuna Lee, Sang Hoon Han, So Young Kwon, Oh Sang Kwon, Sun Suk Kim, Ju Hyun Kim, Yeon Ho Park, Jeong Nam Lee, Soo-Mee Bang, Eun Kyung Cho, Dong Bok Shin, Jae Hoon Lee

**Affiliations:** 1 Division of Hematology and Oncology, Department of Medicine, Gachon Medical School Gil Medical Center, Incheon 405-760, Korea; 2 Division of Gastroenterology, Department of Medicine, Gachon Medical School Gil Medical Center, Incheon 405-760, Korea; 3 Department of General Surgery, Gachon Medical School Gil Medical Center, Incheon 405-760, Korea

## Abstract

**Background:**

Although numerous chemotherapeutic agents have been tested, the role of systemic chemotherapy for hepatocellular carcinoma (HCC) has not been clarified. New therapeutic strategies are thus needed to improve outcomes, and we designed this study with new effective drug combination.

**Methods:**

Twenty-nine patients with histologically-confirmed, metastatic HCC received a combination chemotherapy with doxorubicin 60 mg/m^2 ^and cisplatin 60 mg/m^2 ^on day 1, plus capecitabine 2000 mg/m^2^/day as an intermittent regimen of 2 weeks of treatment followed by a 1-week rest.

**Results:**

The median age was 49 years (range, 32–64) and 19 patients were hepatitis B virus seropositive. Child-Pugh class was A in all patients and 4 had Zubrod performance status of 2. The objective response rate was 24% (95% CI 9–40) with 6 stable diseases. The chemotherapy was generally well tolerated despite one treatment-related death.

**Conclusion:**

Combination chemotherapy with doxorubicin, cisplatin and capecitabine produced modest antitumor activity with tolerable adverse effects in patients with metastatic HCC.

## Background

In Korea, hepatocellular carcinoma (HCC) accounts for 12% of all malignancies and is one of the leading causes of cancer death [1]. Metastatic HCC is associated with a very poor prognosis. Although numerous chemotherapeutic agents have been tested, the role of systemic chemotherapy for metastatic HCC has not been clarified [2,3]. Currently, doxorubicin is the most frequently used drug either in single-agent or in combination [4-6]. Cisplatin also has shown an objective response rate of 17% as single-agent chemotherapy [7], and a higher response rate when combined with epirubicin, 5-fluorouracil (5-FU) [8] or doxorubicin, interferon alpha and 5-FU [9]. Phase II studies have shown that combination chemotherapy with doxorubicin and cisplatin in metastatic HCC patients showed modest antitumor activity with tolerable adverse effects [10,11].

Capecitabine is a rationally designed, orally administered, tumor-selective fluoropyrimidine that mimics continuous infusion 5-FU, which is converted to 5-FU preferentially in tumor tissue by the enzyme thymidine phosphorylase [12,13]. 5-FU is active in patients with HCC [5,6], and most patients clearly prefer oral agents over infusional 5-FU [14]. Furthermore, the low incidence of myelosuppression with capecitabine makes it an attractive agent for incorporation into combination regimens with myelosuppressive agents, such as doxorubicin. It has been reported that mild-to-moderated hepatic dysfunction in patients with colorectal cancer liver metastases did not significantly affect capecitabine pharmacokinetics [15]. Therefore, patients with underlying hepatic dysfunction should be monitored closely during capecitabine treatment, but no dose adjustment solely on the basis of this condition is required.

Based on the demonstrated antitumor activities, different mechanisms of action and toxicity profiles, we designed a phase II study of combination chemotherapy with doxorubicin, cisplatin and capecitabine in patients with metastatic HCC anticipating a synergistic interaction of the combination.

## Methods

### Patients

Patients, who were referred to the department of medical oncology because of their metastatic and/or recurrent HCC, were considered eligible for study entry. They were required to have histologically confirmed, metastatic HCC. All patients had a Zubrod performance status of 2 or lower, a life expectancy of 16 weeks or more, an absolute neutrophil count ≥1,500 mm^-3 ^or more, and a platelet count ≥100,000 mm^-3^. Other inclusion criteria included an adequate renal (serum creatinine level < 2 mg dl^-1^) and cardiac (left ventricular ejection fraction > 50% by echocardiography) function, a Child-Pugh class A or B [16], and a provision of a signed written informed consent. Patients had to have at least one bidimensionally measurable metastatic lesion other than primary HCC. No prior systemic chemotherapy was allowed. This study protocol was reviewed and approved by the Gil Medical Center institutional review board.

### Treatment

The regimen consisted of doxorubicin 60 mg m^-2 ^delivered as an intravenous infusion over 30 min, followed by cisplatin 60 mg m^-2 ^infused over 1 h on day 1. Capecitabine was administered orally at a dose of 1,000 mg m^-2 ^twice daily as an intermittent regimen of 2 weeks of treatment followed by a one-week rest. Each cycle of chemotherapy was given every 3 weeks if the patient's blood count had returned to normal and non-hematologic toxic effects had resolved. Treatment was repeated until disease progression, unacceptable toxicity was detected, or up to a maximum of 6 cycles. Complete blood counts, blood chemistry and serum alpha-fetoprotein (AFP) were obtained before the beginning of each cycle. Dosage of the subsequent cycles was adjusted according to the toxic effects that developed during the preceding cycle. All patients received standard supportive regimen consisting of hydration and antiemetics. No prophylactic administration of hematopoietic growth factors was allowed. However, in cases of persistent viral replication, antiviral treatment (i.e., lamivudine) was permitted.

### Response and toxicity evaluation

Antitumor response was assessed every 2 cycles of chemotherapy and reviewed by an independent investigator later at the time of analyses. The following WHO criteria were used: 1) a complete response (CR) was defined as the disappearance of all radiologic and clinical evidence of tumor and absence of all tumor-related symptoms for at least 4 weeks; 2) a partial response (PR) was defined as a decrease of 50% or more in the product of the 2 greatest perpendicular dimensions of measurable tumors measured at least 4 weeks apart; 3) stable disease (SD) was defined as no significant change in radiologic tumor measurements without no worsening in performance status; and 4) progressive disease (PD) was defined as a decline in performance status, the appearance of new malignant lesions, and an increase of 25% or more in measurable disease. Primary tumor was included for response assessment. Toxicity grading was based on the National Cancer Institute Common Toxicity Criteria version 2.

### Statistical consideration

The primary end-point was the response rate, and the secondary measures were survival and time to progression. Time to progression of disease was calculated from the first cycle of chemotherapy. Survival was defined as the time elapsed from the starting date of chemotherapy to the date of death or the last follow-up. Survival rates and time to progression were assessed by the Kaplan-Meier method. All *P *values were two-sided, with *P *< 0.05 indicating statistical significance. Sample size was calculated to reject a 10% response rate in favor of a target response rate of 30%, with a significance level of 0.05 and a power of 80% by using Simon's optimal two-stage design [17]. In the initial stage, a total of 10 evaluable patients were to be entered and evaluated for response. If more than one response were observed in the first stage, then 19 additional patients were to be entered in the second stage to achieve a target sample size of 29 evaluable patients. Further assessment of the regimen was felt to be warranted if more than five responses were observed in the 29 patients.

## Results

A total of 29 patients were registered in the study between January 2003 and September 2004, and 2 patients were ineligible or never received protocol therapy. However, in an intent-to-treat analysis, these patients were included in the denominator for treatment outcomes. Patient characteristics are summarized in Table 1. The median age was 49 years (range 32–64) and all patients had a good baseline liver function (Child-Pugh class A). All patients had at least one metastatic lesion, with intra-abdominal lymph node, lung, or bone metastases present in 62%, 35% and 38%, respectively. The majority of patients had received prior antitumor therapy. The most common previous treatment for HCC was transarterial chemoembolization, which was received by 16 of 29 patients (55%). Nineteen patients were hepatitis B virus seropositive and 4 had Zubrod performance status of 2.

### Treatment outcomes

**Figure 1:**
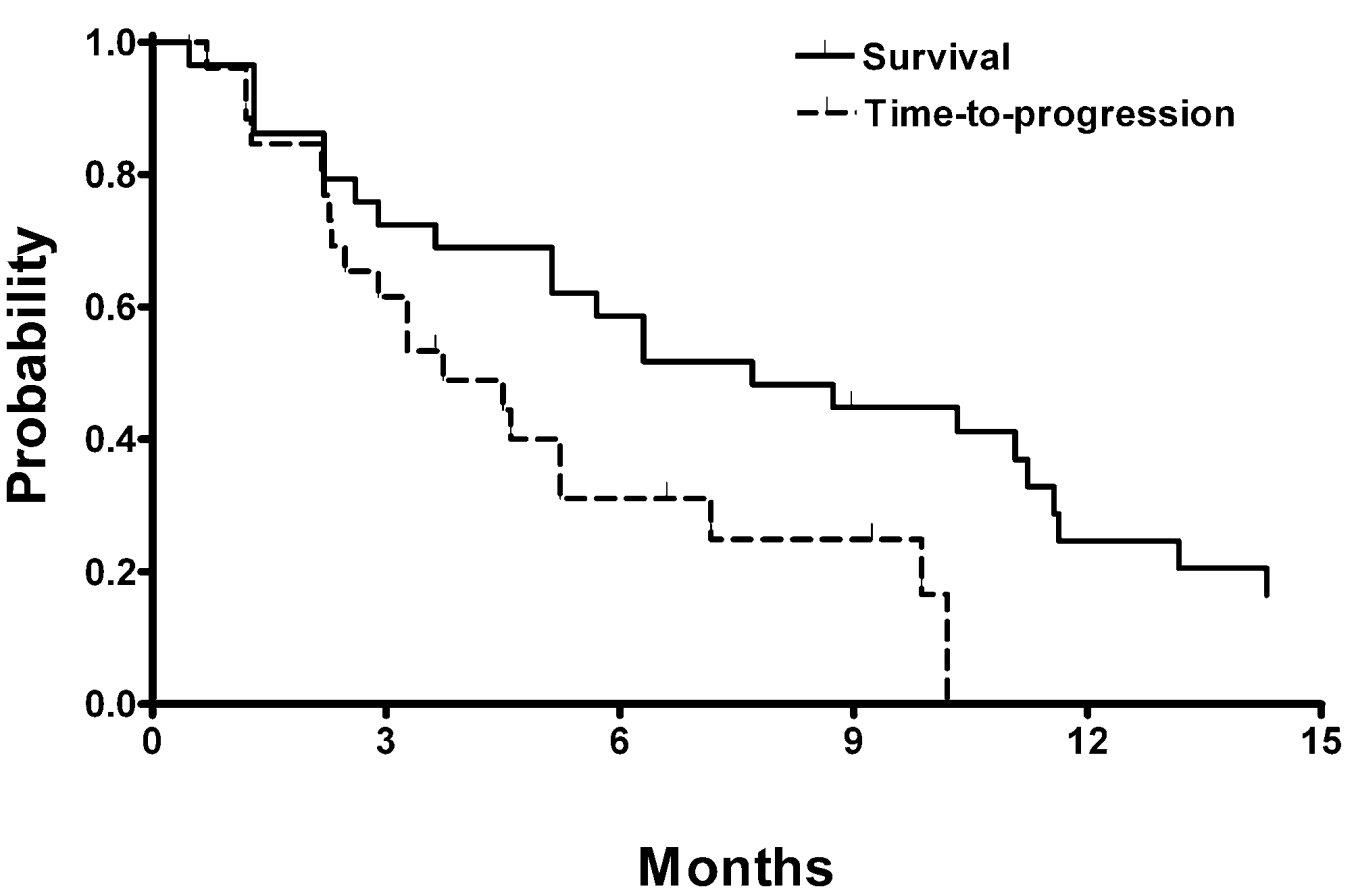
Overall survival and time to progression.

Seven PRs were obtained (24%, 95% confidence interval [CI] 9–40) with 6 SDs. Five patients who had a PR later experienced disease progression. With a median follow-up duration of 17.3 months, the median time to progression of all evaluable patients was 3.7 months (95% CI 1.9–5.6). Median overall survival was 7.7 months (95% CI 3.7–11.7; Figure 1). Five of seven patients who achieved a PR had a corresponding decrease in serum AFP level by more than 50% as compared with the baseline value, and one patient with a PR did not have elevated serum AFP at baseline. Of 25 evaluable patients, 10 (40%) showed more than 50% decrease in serum AFP level from their baseline value and the median time to the AFP response was 1.8 months (range 0.8–4.7). The median survival in patients who showed a reduction in AFP level by 50% was 10.3 months while that in other patients was 3.6 months (*P *= 0.07). Five patients had salvage treatment with thalidomide after completion of chemotherapy.

### Safety

In total, 98 cycles of chemotherapy were administered, with a median of 3 cycles per patient (range 1–6), and 9 (9%) of the planned cycles were delayed because of toxic effects. Dose reduction was required in 17 (17%) cycles. All eligible patients were evaluable for toxic effects (Table 2). The most frequently encountered toxic effects were gastrointestinal toxicities, which were managed with rest, dose reduction, or treatment discontinuation. Only one episode of febrile neutropenia was occurred. Hematologic toxicities were infrequent and no patient received hematopoietic growth factors. Three patients received platelet transfusion during the treatment. Hand-foot skin rash occurred in 17% of patients and was limited to low-grade intensity. Although difficult to differentiate from the symptoms of the underlying disease, severe hepatic dysfunction precluding further chemotherapy was observed in 2 patients. Three patients refused further chemotherapy after the first cycle due to prolonged nausea and/or anorexia. One patient died after the second cycle due to hypovolemic shock which occurred as a complication of gastric variceal bleeding. This event was thought unlikely to be related to the chemotherapy because his platelet counts were differed marginally from pre-treatment values (>100,000 mm^-3^).

## Discussion

HCC often develops among patients who have cirrhosis. It is estimated that approximately 60% of all patients with HCC have underlying cirrhosis [18]. In some patients, cirrhosis associated with portal hypertension and thrombocytopenia makes systemic chemotherapy for HCC extremely difficult and contributes to the poor prognosis associated with HCC. Furthermore, there is no prospective controlled study suggesting that systemic chemotherapy prolongs survival in HCC patients when compared with best supportive care.

Single chemotherapeutic agents, which have demonstrated a consistent response rate of more than 10% are doxorubicin [4,19], 5-FU and cisplatin [5,6]. In a systematic review of clinical trials [3], the 1-year survival rate was superior for doxorubicin-containing regimen over non-doxorubicin-containing chemotherapy. In a study using PIAF regimen (cisplatin, doxorubicin, 5-FU and interferon alpha), 9 out of 50 HCC patients with PR were able to receive surgical resection resulting in pathologic CR in 4 patients [9]. This indicates that aggressive systemic combination chemotherapy may result in CR for HCC patients. However, the PIAF regimen was associated with considerable toxicities with significant myelosuppression, which led to 2 treatment-related deaths. Triplet combination with ECF (epirubicin, cisplatin and 5-FU) was also evaluated in patients with HCC [20]. ECF chemotherapy gave a modest response rate (15%) at the cost of considerable hematologic toxicity.

In an attempt to achieve better tolerability and effectiveness with doxorubicin-containing combination regimen, we substituted 5-FU with capecitabine, which is considered to be as effective and more tolerable than 5-FU [21-23]. Although capecitabine has not been compared to infusional 5-FU to prove similar efficacy, oral administration has the advantage of permitting convenient, patient-orientated therapy, providing the patient with a degree of independence and improved quality of life, while avoiding complications associated with intravenous drug administration. Furthermore, most patients prefer orally administered therapy to intravenous treatment [14]. The presence of mild-to-moderate hepatic dysfunction had no clinically significant effect on the pharmacokinetics of capecitabine and its metabolites [15]. These findings suggest that capecitabine may be useful for patients with HCC [24], including those with impaired hepatic function.

The combination of doxorubicin, cisplatin and capecitabine was well tolerated by patients with metastatic HCC. It should be kept in mind that the degree of cirrhosis was limited to Child-Pugh class A; none of the treated patients had class B or C cirrhosis. Using this triplet combination, we achieved an overall response rate of 24% and a median time to progression of 3.7 months. Forty percent of patients showed a significant reduction in serum AFP in this study. This observation should be interpreted with caution because it represents only a small group of patients with metastatic HCC in this phase II study and the majority of patients had good performance status and preserved hepatic function. As a result, grade 3 or 4 neutropenia occurred only in 14% of patients. This favorable toxicity profile achieved with triplet chemotherapy differed from results reported with other doxorubicin-based chemotherapy for HCC. Although it would be difficult to compare our data directly with the results of other studies, a survival benefit from our triplet combination in HCC seems unlikely, despite the acceptable response rate and favorable toxicity profile observed in our study. The response rate of our regimen is comparable to those found in most phase II studies of doxorubicin-based combination chemotherapy ranging from 13% to 26% [4-6,19,20]. Capecitabine monotherapy was also evaluated in a phase II study, resulting in a modest response rate and good tolerability [24]. Recently, Yeo *et al*. have conducted a phase III study comparing single-agent doxorubicin with PIAF in patients with inoperable HCC [25]. There was no significant difference in response rates and overall survival between doxorubicin and PIAF. To date, none of combination regimens has been recognized as a standard or superior to doxorubicin alone.

With few exceptions, it is generally conceived that systemic chemotherapy is relatively ineffective in HCC [26]. HCC is resistant to chemotherapy because of the high mutational load it carries and the myriads of drug resistance mechanisms [27,28]. This is in addition to the underlying liver dysfunction that imposes lower chemotherapy doses to mitigate toxicity. More recently, targeted therapeutics are being looked at aggressively in HCC. Several potential targets along the angiogenesis and the signal transduction pathway are evaluated in HCC. These include epidermal growth factor receptor, hepatocyte growth factor and its receptor c-met, and vascular endothelial growth factor [29]. Bortezomib, considered as a potent and reversible inhibitor of the proteasome, was also evaluated in a phase I/II study [30]. Interestingly, bortezomib attenuates certain pathways implicating in resistance to anthracyclines, and thus, a significant synergy between bortezomib and anthracyclines was observed [31]. Our next step will be to combine doxorubicin with bortezomib and to investigate the relative place of the targeted therapy in the treatment of metastatic HCC.

## Conclusion

Despite recent advances in our understanding of its genetics and treatment, HCC remains a deadly disease. Although preventive efforts are important, further studies are needed to identify agents that possess more potent activity against HCC. In the current phase II study, the triplet combination of doxorubicin, cisplatin and capecitabine is active chemotherapy in the treatment of metastatic HCC. Further options include additional clinical studies involving novel targeted agents in combination with cytotoxic chemotherapeutics such as doxorubicin.

## Competing interests

The author(s) declare that they have no competing interests.

## Authors' contributions

SHP collected the data, performed the statistical analysis and drafted the manuscript. SYK, OSK, SSK, JHK, YHP, JNL, SB, EKC and JHL followed the patients. DBS designed the study and helped with the manuscript. All authors read and approved the final manuscript.

## Figures and Tables

**Table 1:** Patient characteristics

	No.	%
Total patients enrolled	29	100
Treated	27	93
Age, years		
Median	49	
Range	32–64	
Male gender	24	83
Zubrod performance status		
0	7	24
1	18	62
2	4	14
Child-Pugh classification		
A	29	100
Hepatitis virus serology		
Hepatitis B	19	66
Hepatitis C	0	0
Prior treatment(s)		
Surgery	2	7
Transarterial chemoembolization	16	55
Pretreatment alpha fetoprotein (AFP)		
< 400 ng/mL	8	28
400–10,000 ng/mL	14	48
> 10,000 ng/mL	7	24
Pretreatment laboratory data, mean ± SD		
Bilirubin, mg/dL	0.97 ± 0.34	
Albumin, g/dL	3.17 ± 0.47	
Aspartate transaminase, U/L	71 ± 33	
Alanine transaminase, U/L	42 ± 11	
Platelet, × 10^3^/mL	187.5 ± 54.0	
Metastatic site(s)		
Abdominal lymph node	18	62
Lung	10	35
Bone	11	38

**Table 2:** Worst toxic effects in 29 patients

	Grade 1, 2 (%)	Grade 3, 4 (%)
Anemia	9 (31%)	2 (7%)
Neutropenia	7 (24%)	4 (14%)
Thrombocytopenia	12 (41%)	4 (14%)
Stomatitis	8 (28%)	2 (7%)
Nausea/vomiting	14 (48%)	4 (14%)
Diarrhea	6 (21%)	1 (3%)
Fatigue	6 (21%)	0
Peripheral neutotoxicity	9 (31%)	0
Skin	5 (17%)	0
Hepatic dysfunction	2 (7%)	2 (7%)
Nephrotoxicity	0	1 (3%)
